# Cardioprotective responses to aerobic exercise-induced physiological hypertrophy in zebrafish heart

**DOI:** 10.1186/s12576-021-00818-w

**Published:** 2021-11-08

**Authors:** Zhanglin Chen, Zuoqiong Zhou, Xiyang Peng, Chenchen Sun, Dong Yang, Chengli Li, Runkang Zhu, Ping Zhang, Lan Zheng, Changfa Tang

**Affiliations:** Key Laboratory of Physical Fitness and Exercise Rehabilitation of the Hunan Province, College of Physical Education, Hunan Normal University, No. 529 Lushan South Road, Yuelu District, Changsha, 410012 Hunan China

**Keywords:** Cardioprotective, Aerobic exercise, Physiological cardiac hypertrophy, mTOR signal, Angiogenesis, Mitochondrial homeostasis, Fatty acid oxidation

## Abstract

Herein, we aimed to establish an aerobic exercise-induced physiological myocardial hypertrophy zebrafish (*Danio rerio*) model and to explore the underlying molecular mechanism. After 4 weeks of aerobic exercise, the AMR and *U*_crit_ of the zebrafish increased and the hearts were enlarged, with thickened myocardium, an increased number of myofilament attachment points in the Z-line, and increased compaction of mitochondrial cristae. We also found that the mTOR signaling pathway, angiogenesis, mitochondrial fusion, and fission event, and mitochondrial autophagy were associated with the adaptive changes in the heart during training. In addition, the increased mRNA expression of genes related to fatty acid oxidation and antioxidation suggested that the switch of energy metabolism and the maintenance of mitochondrial homeostasis induced cardiac physiological changes. Therefore, the zebrafish heart physiological hypertrophy model constructed in this study can be helpful in investigating the cardioprotective mechanisms in response to aerobic exercise.

**Supplementary Information:**

The online version contains supplementary material available at 10.1186/s12576-021-00818-w.

## Background

Cardiac hypertrophy is an adaptive response to both physiological and pathological (pressure/volume overload) stimuli, such as exercise and hypertension, respectively, allowing the normalization of the ventricular wall stress [1]. Both types of myocardial hypertrophy involve the enlargement of cardiomyocytes; however, pathological hypertrophy is caused by the extension of a few cardiomyocytes, leading to ventricular cavity dilation and ventricular wall thinning, whereas physiological myocardial hypertrophy is a coordinated increase in the geometric size of cardiomyocytes. Therefore, pathological and physiological stimuli lead to very different outcomes. For instance, pathological stimuli can lead to myocardial fibrosis, systolic dysfunction, and even heart failure [2], whereas physiological hypertrophy can enhance myocardial contractility, increase cardiac output volume, and improve heart function. The understanding of the differences in the mechanisms behind these two types of myocardial hypertrophy is thus, of great clinical significance. Of note, while pathological hypertrophy, as common heart disease, has been extensively studied [3, 4], the molecular mechanisms behind exercise-induced physiological hypertrophy are mostly unknown.

Recently, exercise interventions have been explored for the prevention and treatment of cardiovascular diseases. Although the underlying mechanisms are still unclear [5], an appropriate amount of regular aerobic exercise can effectively prevent or even treat cardiovascular diseases characterized by pathological hypertrophy, including hypertension and myocardial infarction [6]. Interestingly, it was found that aerobic exercise could reduce the risk of heart disease; this effect was not inferior to that of pharmacotherapy [7]. Of note, in healthy people with regular exercise habits, the physiological hypertrophy of the heart is called “athlete’s heart.” Indeed, cardiac hypertrophy in response to exercise is generally considered protective; in some cases, it can improve heart function and does not develop into heart failure. However, Alisson et al. [8] found that overtraining can lead to the high expression of proinflammatory factors in cells with the potential to induce pathological changes in the heart. Indeed, evidence suggested the link between exercise and expression of proinflammatory factors may be ‘dose-dependent’ [9], the adjustment of the intensity of exercise needs to achieve the intended beneficial training outcome. Therefore, the study of reasonable exercise programs for the induction of physiological heart hypertrophy and the elucidation of the regulatory mechanisms behind is essential; valuable monitoring indicators of athletes’ myocardial hypertrophy can be disclosed and exercise interventions can be developed for the treatment of pathological myocardial hypertrophy.

The zebrafish genome is remarkably similar to the human genome [10]. In recent years, a large number of studies have used the zebrafish heart as a model of the mammalian heart; a high similarity between the zebrafish and human hearts in terms of the heart rate, action potential duration, and morphology is also observed [11]. Importantly, zebrafish are associated with the ease of genetic manipulation, as well as rich developmental bioinformatics, and are suitable for a variety of experimental techniques [12] and are migratory, so the device can be used to train zebrafish in non-invasive swimming exercises. This all facilitates the kinematic study of skeletal and cardiac muscles [13].

Here, first, we constructed a zebrafish model of physiological cardiac hypertrophy induced by aerobic exercise. The model presents cardiac hypertrophy, compact myocardium thickness and a cross-sectional area of spongy myocardium, and mitochondrial cristae thickening. Concurrently, we explored the mechanism behind the development of physiological myocardial hypertrophy. The expression of genes related to the IGF1/PI3K/AKT1/mTOR axis, mitochondrial biogenesis, and selective autophagy was upregulated, and lipid metabolism and the antioxidant capacity of cardiomyocytes were enhanced. This model can provide a research foundation for the study of exercise in the context of the prevention/treatment of cardiovascular diseases, and the cardioprotective mechanisms unraveled in this study will provide guidance for the monitoring of the health of the heart, and the adjustment of the intensity of exercise.

## Methods

### Animal care and ethics

The experimental fish used in this study were purchased from the National Zebrafish Resource Center, the Chinese Institute of Aquatics, and were raised in the zebrafish laboratory of the Hunan Provincial Key Laboratory of Physical Fitness and Sports Rehabilitation. This study was approved by the Experimental Animal Use Ethics Committee of Hunan Normal University (Ref. No. 2018-046). We used the same batch of healthy male zebrafish (*Danio rerio*; wild-type AB strain) at the age of 8 months [body length (BL): 2.588 ± 0.0326 cm; body weight (BW): 0.25 ± 0.013 g; *n* = 45]. Fish were fed with fresh Artemia. The conditions of the breeding water were monitored daily. The water temperature, conductivity, and pH were maintained at 28 ± 1 °C, 550 ± 50 μs/cm, and 7.5 ± 0.3, respectively; in addition, animals were maintained under light/darkness cycles of 14/10 h. The experimental fish were randomly divided into two groups: the sedentary control group (Control, *n* = 20) and the aerobic exercise group (Exercise, *n* = 20). Fresh brine shrimps were provided to fish before and after exercise and as a supplement at 9 p.m.; control fish were equally fed.

### Determination of the critical swimming speed (*U*_crit_), active metabolic rate (AMR), and optimal swimming speed (*U*_opt_)

*U*_crit_ is the maximum continuous swimming velocity, the most important indicator of the “sports performance” of zebrafish [14]. AMR, which is the highest metabolic rate the fish can sustain under maximal activity and can be used to measure the exercise metabolism of zebrafish [15, 16].

Prior to the formulation of an exercise program, it is necessary to measure the AMR and *U*_crit_ to calculate the *U*_opt_ required for aerobic exercise [17]. Before the experiments, the experimental fish were anesthetized with 40 mg/L MS-222 (using culture water), and their standard BL (cm) and BW (g) were measured.

Swimming ability tests and determination of oxygen consumption was carried out in a variable-speed miniature swim tunnel respirator equipped with a DAQ-BT control device and the AutoRespTM software (Loligo Systems, Tjele, Denmark). The system includes a 170 mL closed swimming tunnel, submerged in a 20 L buffer tank used to supply 28 ± 0.5 °C oxygen-rich circulating water. The concentration of oxygen (O_2_) is measured with a fiber-optic oxygen immersion probe connected to a Witrox1 miniature oxygen meter sensor (Loligo Systems).

The fish were fasted 24 h prior to AMR and *U*_crit_ analyses. Only one experimental fish is tested at a time in the swimming tunnel. To stabilize the adult zebrafish to the minimum metabolic rate after introduction into the swimming tunnel respirometer, the individual experimental fish were allowed to adapt for 1.5–2 h at a minimum water velocity of 0.8 BL/s. To determine the *U*_crit_, the swimming speed of the fish was set to increase gradually (2.7 BL/s every 7 min through a DAQ-BT speed control system), and the same speed level was tested twice; the measurements were carried out at the same water flow velocity increment for 14 min. Each 7 min loop consisted of a 5 min measuring phase followed by a 1.5 min flushing phase and a 0.5 min waiting phase. The determination was concluded when the fish were exhausted, i.e., unable to keep swimming at the current water speed and, as a result, unable to move from the rear screen after being washed for 20 s. *U*_crit_ was defined as per the Brett calculation formula [18]:

1 $$ U_{{{\text{crit}}}} = U_{{\text{f}}} + U_{{\text{S}}} \times \left( {T_{{\text{f}}} /T_{{\text{S}}} } \right), $$

where *U*_f_ (cm/s) is the maximum swimming velocity reached before exhaustion, *U*_S_ (2.7 BL/s) is the velocity increment, *T*_f_ (min) is the time at the maximum swimming velocity before exhaustion, and *T*_S_ (14 min) is the duration of each velocity increment.

The AMR was measured simultaneously, using the automatic intermittent flow respiration method; AMR was calculated in the background using the AutoRespTM software. Briefly, the AMR was measured twice in each water flow velocity increment, and the average value of the two AMRs was used for statistical analysis. AMR is defined as the amount of oxygen consumed at each increment of water flow velocity in the *U*_crit_ test.

The cost of transport (COT) (J/kg/m) was then calculated via the division of AMR (mmolO_2_/kg/h) by the corresponding swimming velocity *U* (m/s) [15]. A polynomial curve was drawn to record the COT and the swimming speed of each fish to calculate the lowest COT (COTmin) point on the curve, which is at *U*_opt_ of the experimental fish. The obtained formula is as follows:

2 $$ {\text{COT}} = 0.271 + 0.002U^{2} - 0.037U\left( {R^{2} = 0.98} \right), $$

where *U* is the increasing speed.

### Exercise regimen and motion device

Via the substitution of the tested speed into the above polynomial Eq. (2), the lowest COT of the experimental fish was determined as 23.68 ± 0.83 cm/s (*U*_opt_). Before determining the exercise regimen, we trained zebrafish under 80%, 100%, and 120% *U*_opt_, found that after 100% *U*_opt_ training, zebrafish cardiac mTOR signal was enhanced, and the mRNA expression of pathological marks such as *myl7* and *nppa* was relatively low (Additional file 1: Fig. S1). Therefore, 100% *U*_opt_ was used as the aerobic swimming velocity for training. The experimental fish were fed at 9:30 in the morning every day, and the amount of feeding was controlled to allow a 10 min resting period before training. The fish were then transferred into a zebrafish exercise device (Additional file 1: Fig. S2) designed by our laboratory: a 90 cm long, 25 cm wide, and 30 cm high chamber containing a variable-frequency power pump and a swimming tunnel of 52 cm long and 6 cm in diameter. This device is sufficient for the simultaneous exercise training of 20 experimental fish. During exercise, the water temperature was maintained at 28 °C (similar to the rearing temperature) using a temperature control system, and the oxygen supply was ensured in real-time using an air pump. The aerobic exercise group started to adapt for 10 min at a speed of 5 cm/s every day; the speed was then gradually increased to 23.5 cm/s and the fish were allowed to exercise for 4 h (10:00–14:00). Fish exercised for 6 days and rested for 1 day for a total of 4 weeks. The control group was put into another identical system but without the exercise intervention at the same time. At the experimental endpoint, fish were treated with 0.25 mg/mL MS-222 anesthetic to relieve pain before their hearts were removed. Hearts to be morphologically analyzed were placed in 1× PBS, and then the others hearts with excess tissue removed were fixed in 4% paraformaldehyde fixative. The others were placed in liquid nitrogen.

### Hematoxylin & eosin (H&E) staining of the heart sections

**Fig. 1:**
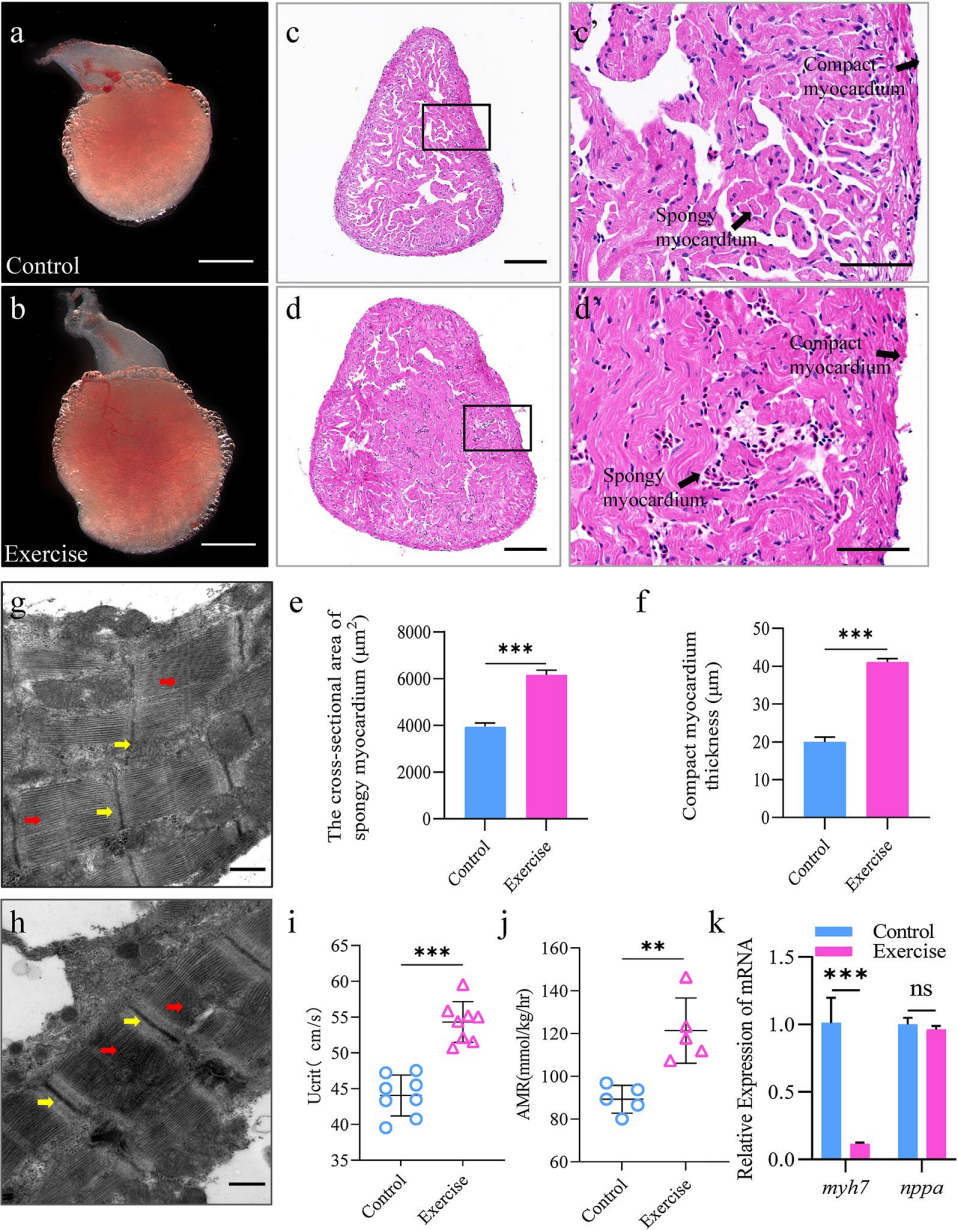
Exercise induces physiological cardiac hypertrophy in zebrafish. **a**, **b** Representative images of the overall appearance of the hearts from zebrafish in the control (**a**) and exercise (**b**) groups; scale bar = 1 mm; **c**, **d** representative H&E-stained cross-sections of the hearts from zebrafish in the control (**c**) and exercise (**d**) groups; scale bar = 50 μm. **c′**, **d′** Magnified images of the black boxes in **c**, **d**, representing the compact and spongy myocardium; scale bar = 60 μm. **e** Zebrafish compact myocardium thickness and **f** cross-sectional area of spongy myocardium per group. **g**, **h** Representative transmission electron micrographs of heart tissues from zebrafish in the control (**g**) and exercise (**h**) groups; the yellow arrows highlight the Z lines; the red arrows indicate the myofilaments; scale bar = 500 μm. **i**, **j** Critical swimming speed (*U*_crit_) and the active metabolic rate (AMR) increased with continuous exercise. **i** *U*_crit_ was determined in animals from the sedentary control and exercise groups (*n* = 8). **j** AMR was measured in animals from the sedentary control and exercise groups (*n* = 5). Each symbol represents an animal. The error bars represent the standard error of the mean. Statistical significance was determined using the Student’s *t* test: **p < 0.01; ***p < 0.001. **k** mRNA levels of the pathological hypertrophy indicators *myh7* and *nppa* in the heart per group. The error bars represent the standard error of the mean. Statistical significance was determined using the Student’s *t* test: *ns* not significant, ***p < 0.001. *H&E* hematoxylin & eosin

Hearts were dehydrated, cleared in xylene, and embedded in paraffin. Continuous paraffin sections of 4 µm were cut using an RM2016 microtome (Leica, Wetzlar, Germany) and mounted on slides (Servicebio, Wuhan, China). H&E-stained images were acquired with a NIKON DS-U3 camera control unit (Nikon, Tokyo, Japan). The quantification of the compact myocardium thickness and cross-sectional area of spongy myocardium was calculated as shown in Fig. 1 and was averaged in the context of sections for 3 hearts. The average thickness of the compact layer from five sections of each heart at four random locations was determined using ImageJ software [19].

### Quantitative real‐time PCR (qPCR)

Total RNA from the heart tissues was extracted with the TRIzol™ Reagent (Ambion, Thermo Fisher Scientific, Waltham, MA, USA). A micro-concentration detector was used to measure the RNA concentration and purity; only samples with A260/A280 values between 1.8 and 2.0 were used. One microgram of total RNA per sample was then reversely transcribed using the PrimeScript™ RT reagent Kit with gDNA Eraser (Takara, Kyoto, Japan) in a total volume of 20 μL to obtain cDNAs. qPCR was then performed in 10 μL reactions, including 5 μL of 2× PowerUp SYBR Green Master Mix (Thermo Fisher Scientific), 2 μL of cDNA template, 0.3 μL of each primer (10 nM forward and reverse primers), and water. The reaction was run in a BioRad CFX ConnectTM Optics Module (BioRad, Hercules, CA, USA) as follows: pre-denaturation at 95 °C for 5 min, 40 cycles of 95 °C for 30 s and 60 °C for 1 min, and melting curve determination from 60 to 95 °C (0.5 °C increments every 10 s). The fluorescence values in each well were recorded in real time and used to calculate the threshold cycle (CT). The list of genes studied and the respective primer sequences are shown in Additional file 1: Table S1. Gene expression was defined as follows: the CT value of each target gene was normalized with that of the internal reference gene (either *gapdh* or *cox4il*, the latter for mitochondrial genes) and then the 2^−ΔΔCT^ method was used to calculate the expression of each target gene.

### Western blotting

The experimental fish hearts were lysed in RIPA buffer (high) (Solarbio, Wuhan, China) containing 1 mM phenylmethylsulfonyl fluoride (PFMS), 1× protease phosphatase inhibitor (Solarbio, Wuhan, China). Quantitative the total protein by BCA Protein Quantification Kit (Vazyme, Nanjing, China). 20 μg of protein lysates were loaded in the SDS-PAGE gel, and after electrophoresis, the PAGE gel was cut off according to the molecular weight of the target protein, and the protein was transferred to the PVDF membrane. Membranes were blocked at room temperature using 5% skim milk diluted in PBST for 2.5 h and probed overnight at 4 °C with the primary antibody. Membranes were washed in PBST 3 times for 10 min each and then incubated for at least 2 h at room temperature with goat anti-rabbit IgG-HRP secondary antibody (1:10,000, Absin, Shanghai, China). Acquire image using darkroom development techniques for chemiluminescence, Perform ECL as described by the manufacturer, add ECL reagents (Servicebio, Wuhan, China) for 1–2 min at RT and capture WB image using various durations of exposure. Primary antibodies used were: GAPDH (1:2000, Servicebio Wuhan, China), AMPK Alpha 1 (1:1000, Proteintech, Wuhan, China), SIRT1 (1:2000, Abcam, UK), PI3K (1:2000, Proteintech, Wuhan, China), AKT1 (1:2000, CST, USA), FIS1 (1:3000, Proteintech, Wuhan, China), OPA1 (1:3000, Proteintech, Wuhan, China), MFN2 (1:3000, Proteintech, Wuhan, China), LC3 (1:1000, Abcam, Cambridge, UK). Immunoreactive bands were visualized by a Chemiluminescent gel imaging system (Tanon 5200Multi, Shanghai, China) and quantified with ImageJ software (Additional file 1: Figs. S4–S6).

### Statistical analysis

The GraphPad Prism 8 software (GraphPad Software Inc., San Diego, CA, USA) was used for data representation and statistical analysis. All data are normally distributed and expressed as the mean ± standard error of the mean. Significance was determined using the one-way analysis of the variance or the unpaired sample *t* test. Statistical significance was determined by p values lower than 0.05; the symbols *, **, and *** represent *p* < 0.05, *p* < 0.01, and *p* < 0.001, respectively, *versus* the control group; ns represents *p* > 0.05, without statistical significance. All experiments were repeated at least three times.

## Results

### Exercise training generates a physiological cardiac hypertrophy zebrafish model

We investigated if 4 weeks of aerobic exercise training would induce cardiac hypertrophy in zebrafish. At the experimental endpoint, the BL and BW of zebrafish in exercise group showed upward trends compared with those of the control group, but the difference was not statistically significant (Additional file 1: Fig. S3a, b). Next, the hearts of zebrafish were observed using a stereomicroscope. As shown in Fig. 1a, b, the hearts of the exercise group were significantly enlarged. The tissues were further stained with H&E, and the cross-sectional area of the heart, the thickness of the compact myocardium, and the cross-sectional area of spongy myocardium of the hearts of the zebrafish in the exercise group were found to be significantly increased (Fig. 1c–f). Moreover, transmission electron microscopy further revealed that the myofibrillar density of cardiomyocytes in the exercise group increased with the number of myofilament attachment points in the Z-line (Fig. 1g, h), suggesting an enhancement in contractile force. To investigate changes in the exercise capacity of zebrafish after training, we measured the *U*_crit_ and AMR. Importantly, both indicators were significantly higher in zebrafish subjected to exercise than those in the control group (Fig. 1i, j). To further identify the type of cardiac hypertrophy in zebrafish after 4 weeks of aerobic exercise training, we performed qPCR to evaluate the expression levels of the known heart disease markers *nppa* and *myh7*. Importantly, there was no difference in the mRNA levels of *nppa* between the exercise and control groups (Fig. 1k). However, the expression levels of *myh7* were significantly lower in the exercise group (Fig. 1k). The above results suggest that after 4 weeks of aerobic exercise intervention, zebrafish develop a phenotype of physiological cardiac hypertrophy.

### Exercise training activated the mTOR signal

**Fig. 2:**
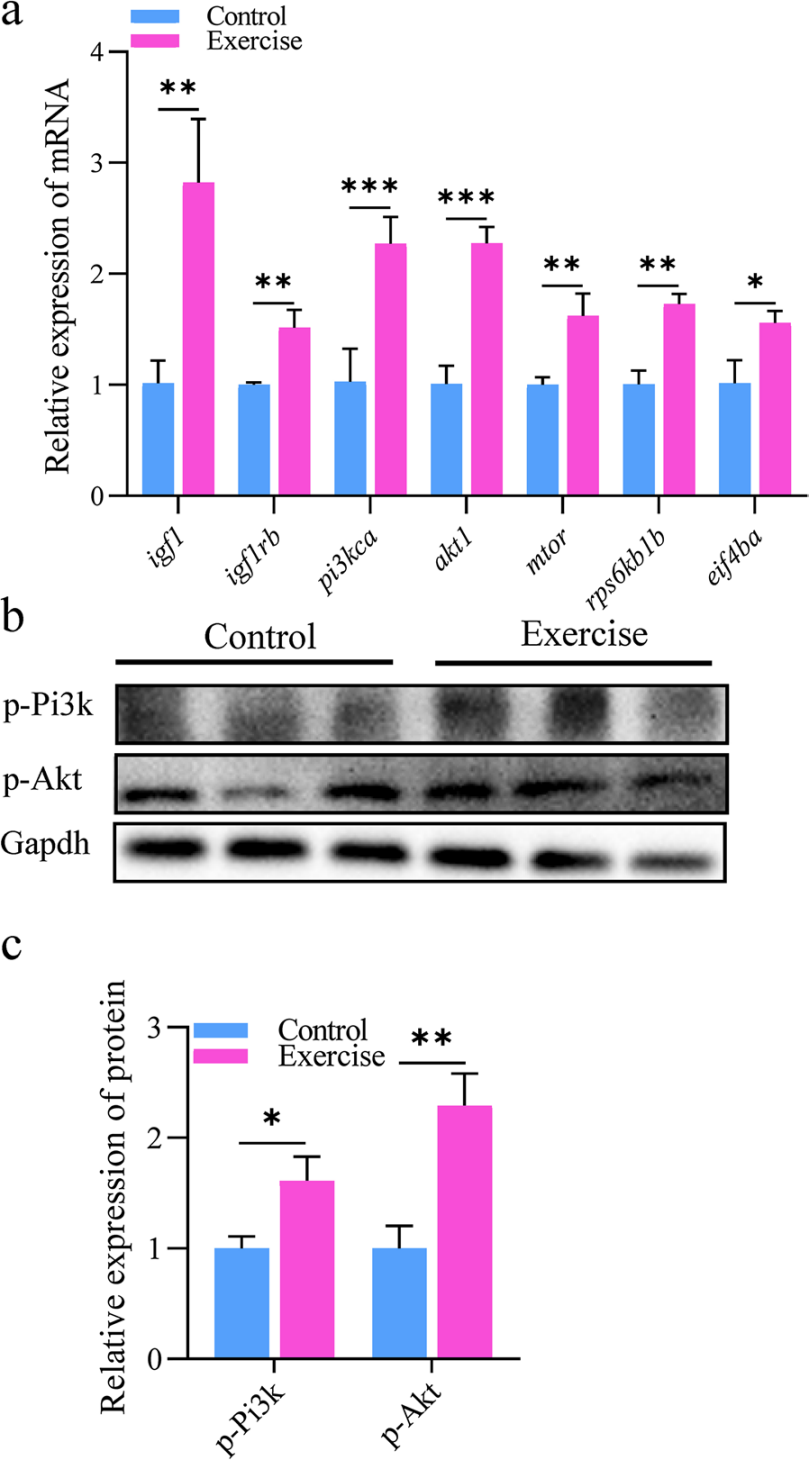
PI3K-AKT signaling is activated after aerobic exercise training. **a** mRNA levels of IGF1/PI3K/AKT1/mTOR signaling-related genes (essential for protein synthesis) in the hearts of zebrafish. **b**, **c** Protein expression of Pi3k and Akt for protein synthesis in the hearts of zebrafish. The error bars represent the standard error of the mean. Statistical significance was determined using the Student’s *t* test: *p < 0.05; **p < 0.01; ***p < 0.001

The significant enlargement of the heart and the evident thickening of the ventricular wall after exercise training implied an increase in the protein turnover in cardiomyocytes. The mTOR pathway is the key signaling pathway that promotes protein synthesis in hearts with physiological hypertrophy [20]. Therefore, we detected the expression of mTOR signal pathway-related factors in the zebrafish heart. We found that the mRNA levels of *mtor* and its upstream and downstream targets, such as *igf1*, *igf1rb*, *pik3ca*, *akt1*, *rps6kb1b*, and *eif4ba*, were significantly increased in the exercise group compared with those in the control group (Fig. 2a). Moreover, we found an increase in the phosphorylation of PI3K-AKT, the upstream activation signal for mTOR (Fig. 2b, c), suggesting that the cardiac mTOR signal was activated by this aerobic training program.

### Exercise training promoted angiogenesis

**Fig. 3:**
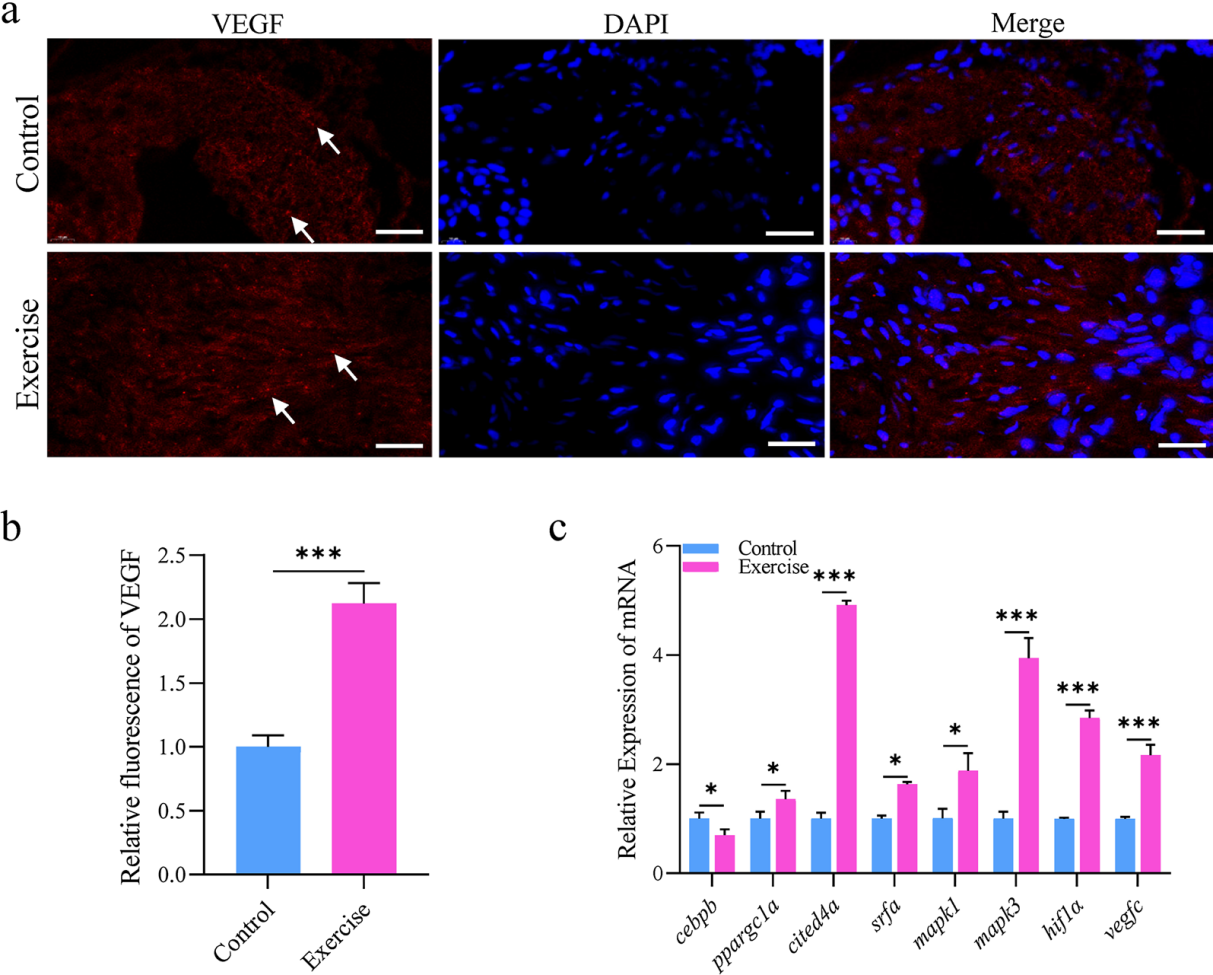
Aerobic exercise training programs promoted angiogenesis. **a** Staining of cardiac vessels for vegf in both exercise and control groups; the white arrows refer to the positive staining of blood vessels in the heart of zebrafish, scale bar = 20 μm. **b** Relative fluorescence of vegf for the two groups analysed by ImageJ. **c** mRNA expression of genes related to angiogenesis in zebrafish heart. Statistical significance was determined using the Student’s *t* test: ***p < 0.001, *ns* not significant

Sufficient new capillary density to support oxygen transport, nutrient delivery, and the discharge of metabolic waste of cardiomyocytes is the morphological feature of physiological hypertrophic heart [21, 22]. To detect the angiogenesis of the hypertrophic model in this experiment, both groups were immuno-stained with vascular endothelial growth factor (VEGF). The results showed that vegf expression was significantly increased after aerobic exercise (Fig. 3a, b). Consistently, we found that the mRNA expression of angiogenic factors such as *cebpb*, *ppargc1a*, *cited4a*, *srfa*, *mapk1*, *mapk3*, *hif1ab*, and *vegfc* was significantly increased (Fig. 3c). Altogether, the above results indicate that aerobic exercise-induced physiological cardiac hypertrophy in zebrafish is accompanied by the formation of new blood vessels.

### Aerobic exercise induces the dynamic changes in mitochondrial morphology

**Fig. 4:**
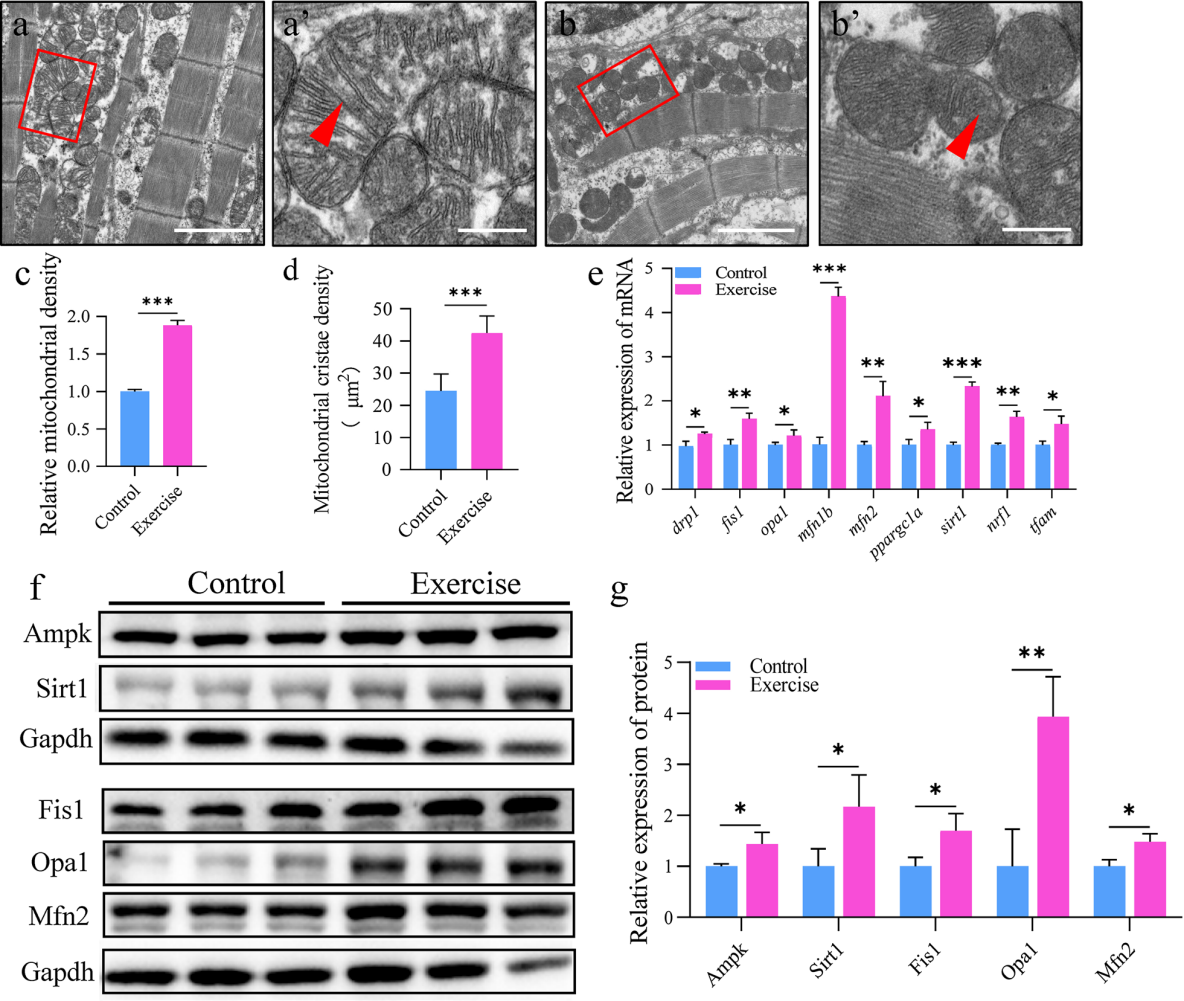
Aerobic exercise induces dynamic changes in mitochondria morphology. **a**, **b** Representative transmission electron micrographs of cardiomyocytes from the control (**a**) and exercise (**b**) groups; scale bar = 2 μm. **a′**, **b′** Magnified images of the red boxes in **a**, **b**, representing the mitochondria; red triangles point to the mitochondrial cristae; scale bar = 0.4 μm. **c** Relative mitochondrial density. **d** Mitochondrial cristae density. **e** mRNA expression of mitochondrial fission and fusion-related genes in zebrafish heart. **f**, **g** Protein expression for fission and fusion in the hearts of zebrafish. Statistical significance was determined using the Student’s *t* test: ***p < 0.001

The increase in the *U*_crit_ and AMR of zebrafish indicates enhanced energy metabolism in the heart. Mitochondria are the most important subcellular organs for ATP production and adapt their shape to sustain necessary cellular functions [23]. We used transmission electron microscopy to examine the mitochondria in detail and measured the density of mitochondria and mitochondrial cristae according to the method of Nielsen et al. [24]. The results showed that the density of mitochondria increased, and mitochondrial cristae were more compacted, with decreased matrix content, after training (Fig. 4a–d). Mitochondria modulate their functions and status via mitochondrial fusion and fission events [23]. We found that the mRNA expression of the genes involved in mitochondrial fusion and fission, such as *drp1*, *fis1*, *opa1*, *mfn1b*, and *mfn2* (Fig. 4e), as well as that of the proteins Fis1, Opa1, and Mfn2 (Fig. 4f, g), was upregulated, suggesting that mitochondrial fusion–fission cycle increased in response to aerobic exercise. It is reported that exercise activates the signaling pathways that converge to initiate mitochondrial biogenesis. Indeed, the regulators of mitochondrial biogenesis, *ppargc1a/PGC-1α*, *sirt1*, *nrf1*, and *tfam* (Fig. 4e), were detected to be upregulated in the heart of the exercise group, as well as the upstream AMPK and SIRT1 proteins were both upregulated (Fig. 4f, g).

### Mitochondrial homeostasis in response to aerobic exercise

**Fig. 5:**
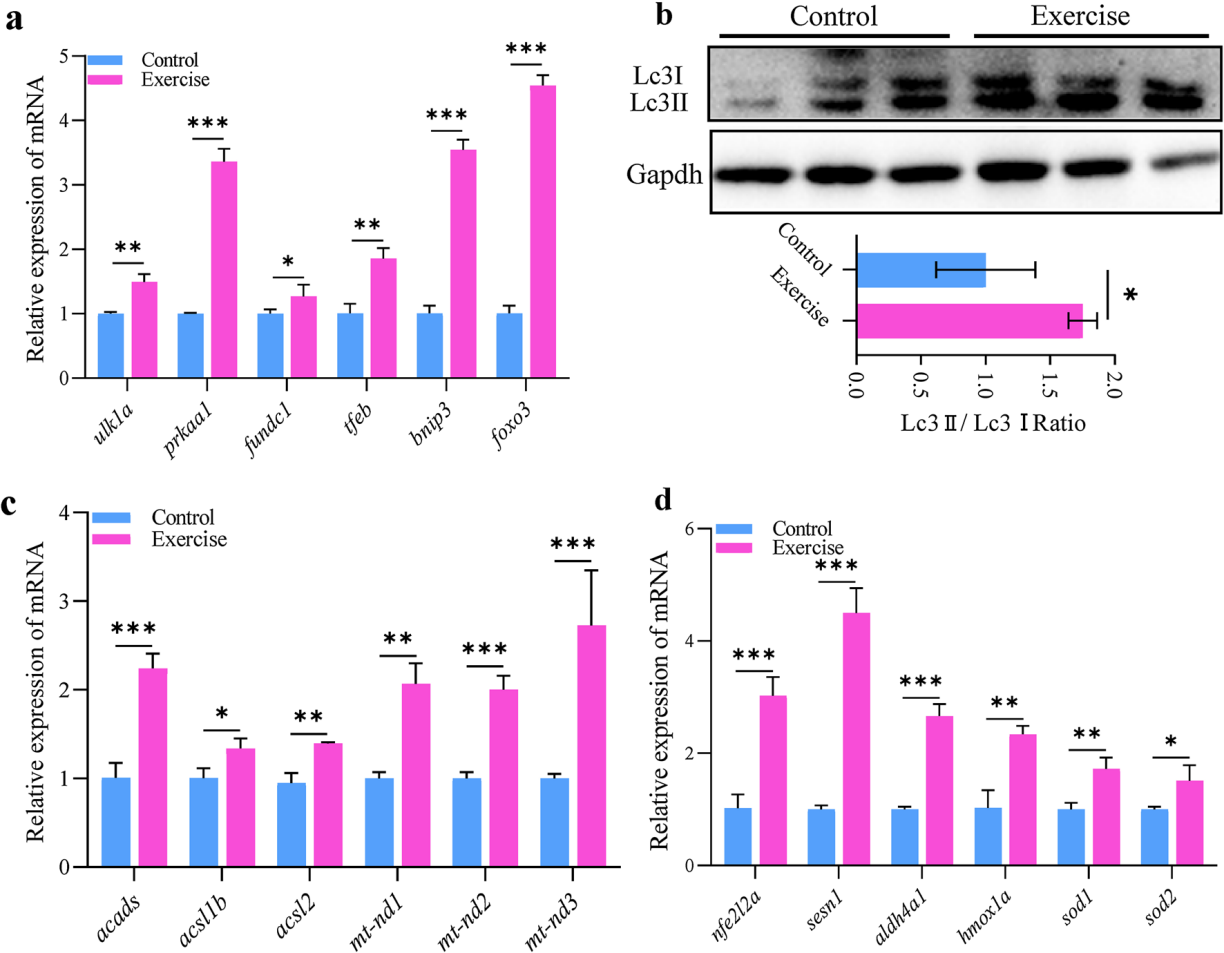
Mitochondrial homeostasis in response to aerobic exercise. **a** mRNA expression of mitochondrial autophagy markers in the hearts of zebrafish. **b** Protein expression of Lc3 I and II for autophagy in the hearts of zebrafish. **c** mRNA expression of lipid metabolism- and electron transport complex I-related genes in the hearts of zebrafish. **d** mRNA expression of antioxidant machinery-related genes in the hearts of zebrafish. Statistical significance was determined using the Student’s *t* test: *p < 0.05; **p < 0.01

Mitophagy, as a cell-protective process to remove dysfunctional mitochondria, is sensitive to energy stress and is activated in response to acute endurance exercise [25]. We found that the sensor of energy, AMPK (Fig. 4f, g), and its downstream target ULK1, which are the marks in exercise-induced mitophagy, was upregulated in heart of the training group (Fig. 5a). The mRNA expression of mitophagy-related genes, such as *prkaa1/AMPK*, *fundc1*, *tfeb*, *bnip1*, and *foxo3* (Fig. 5a), also increased with increased LC3-II/LC3-I ratio (Fig. 5b), implying an elevation in basal mitophagy.

Under starvation, an activated AMPK signal can induce fatty acid oxidation in mitochondria to provide energy supply. In the heart of aerobic exercise zebrafish, the expression of genes involved in fatty acid catabolism, such as *acads*, *acsl1b*, and *acsl2*, and components of the complex I of the electron transport chain, such as *mt-nd1*, *mt-nd2*, and *mt-nd3*, was upregulated (Fig. 5c), suggesting physiological hypertrophy induced by exercise stimuli might co-ordinately regulate fatty acid oxidation and mitochondrial oxidative capacity.

However, excessive oxidation will lead to the accumulation of ROS, which is harmful to cell homeostasis. To explore the mechanism of cell resistance to oxidative stress, we detected the expression of genes related to the antioxidant response, such as *nfe2l2a*, *sesn1*, *aldh4a1*, *hmox1a*, *sod1*, and *sod2*, by qPCR and found that they were all upregulated (Fig. 5d). These results indicate that cardiomyocytes adapt to the energy demands of aerobic exercise through physiological changes, such as in the rates of fatty acid metabolism and antioxidation.

## Discussion

**Fig. 6:**
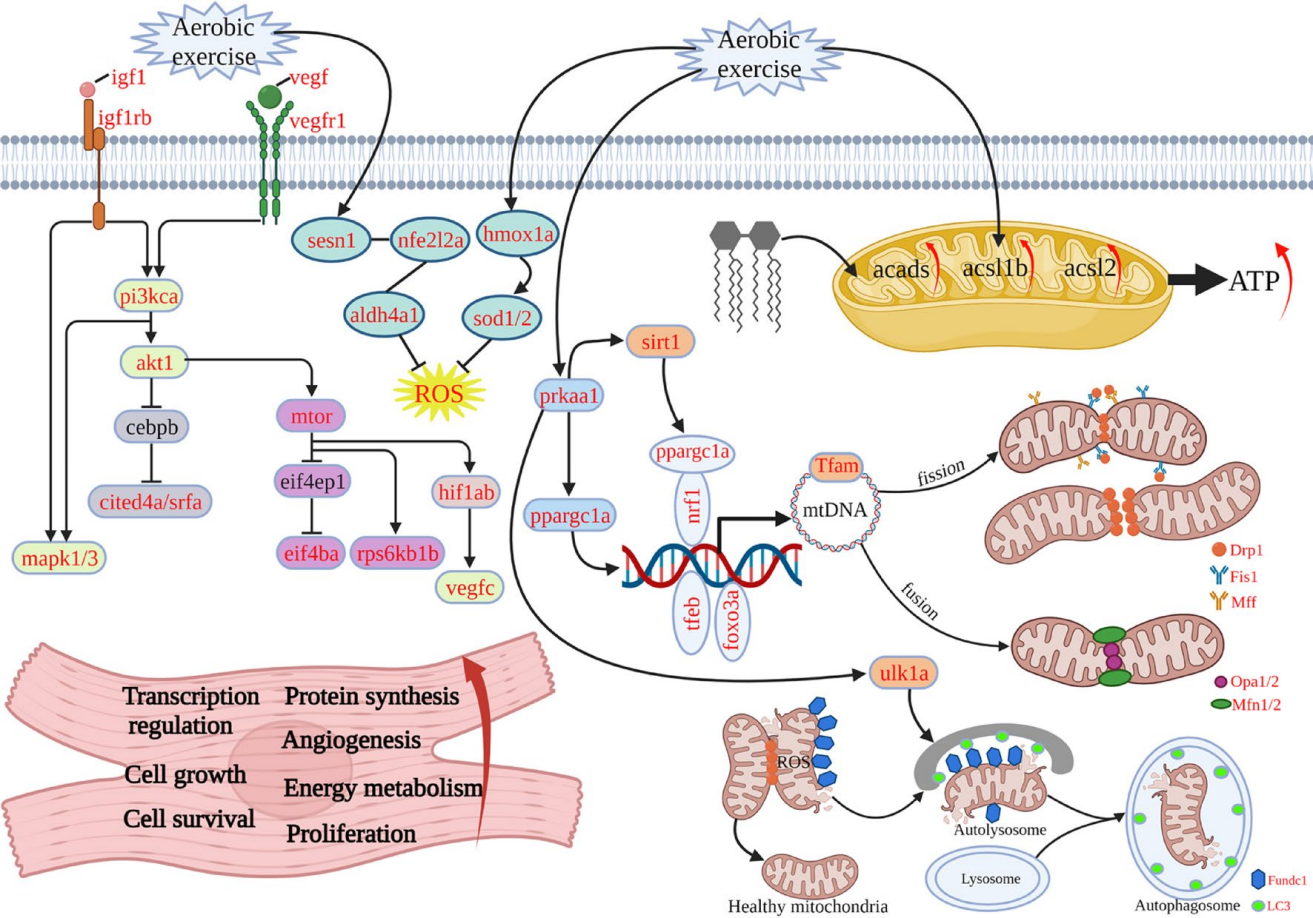
Molecular mechanism of post-exercise regulation of cardiac hypertrophy in zebrafish. (1) Due to the requirement of a large amount of energy supply under training conditions, the mitochondria need to keep homeostasis between energy production and consumption. (2) Mitochondrial function enhancement: aerobic exercise also activated prkaa1-pargc1a signaling, which enhanced mitochondrial biogenesis, division, fusion, and autophagy processes, thus improving mitochondrial function. (3) Elevated cardiac energy metabolic function: a large amount of energy is consumed during aerobic exercise. acads, acsl1b, and acsl2 lipolytic metabolizing enzymes are also activated for expression; (4) improve antioxidant capacity: as exercise generates ROS, excessive ROS can cause damage to organelles and DNA. At the same time, antioxidant genes such as HO1-SOD and SESN1 are activated to scavenge ROS (Created with BioRender.com)

Adaptive structural and physiological changes provide the heart with strong plasticity to improve its function and meet the increasing demands for oxygen convection, nutrient supply, and metabolic waste discharge in response to external stimuli. After aerobic exercise intervention, in this study, zebrafish heart showed increased cross-sectional area, ventricular wall thickening, and *U*_crit_ and AMR, suggesting an adaptive protective response. Further immunobiological and molecular investigation indicated that the protective effect of the cardiac adaptive response may be closely related to mTOR signaling, angiogenesis, and mitochondrial homeostasis (Fig. 6).

Although both physiological hypertrophy and pathological hypertrophy initially develop as an adaptive response to cardiac pressure, they differ greatly in molecular mechanisms, cardiac histomorphology phenotype, and prognosis. Physiological cardiac hypertrophy is characterized by a mild increase in cardiac mass and increased ventricular volume, with a coordinated increase in wall thickness and enhancement of cardiac function. Conversely, pathological hypertrophy progresses to ventricular chamber dilatation with wall thinning through lengthening of individual cardiomyocytes, contractile dysfunction, and heart failure. In this study, the zebrafish from the aerobic training groups showed physiological heart hypertrophy, with increased cross-sectional area, thickened ventricular wall, and increased *U*_crit_ and AMR. Although *U*_crit_ and AMR have a strong association with cardiac function, other influencing factors, such as the functional improvement in the skeletal muscle, lungs, and liver, may also contribute to the increment of *U*_crit_ and AMR [17, 26, 27]; this would be an interesting topic for future investigations.

The ventricular wall thickening of physiological hypertrophy accompanied by increased protein turnover is generally considered to be related to the activation of the mTOR signaling pathway [20]. It is reported that IGF1 and IGF1 receptor (IGF1R) combine to activate the intracellular PI3K/AKT1/mTOR phosphorylation cascade, and finally activate rps6k and eif4be, to increase the protein synthesis of cardiomyocytes and promote the physiological hypertrophy of the heart [28]. In the model of exercise-induced physiological hypertrophy in mice, the expression of IGF1, IGF1R, PI3K, AKT1, mTOR, S6K, and EIF4ea was upregulated, indicating that PI3K/AKT1/mTOR signal pathway was activated by IGF1 signal [28, 29]. In this study, we found that the mRNA expression levels of mTOR upstream activators igf1, igf1r, and downstream effectors rps6k and eif4be were significantly upregulated, and the phosphorylation of PI3K/AKT1 protein was significantly increased, indicating that this exercise training program stimulated the activation of cardiac mTOR and promoted protein synthesis in cardiac myocytes. Rovira et al. [30] found that the *U*_opt_ regimen can increase cardiomyocyte proliferation under regenerating conditions. After exercised at *U*_opt_ for 6 h/day for 5 days/week in a total of 20 experimental days over 4 weeks, the proliferative capacity of cryoinjured zebrafish heart was increased but the hypertrophic response was not activated. In our study, the zebrafish were trained to swim with *U*_opt_ for 4 h/day for 6 days/week over 4 weeks. Under the stimulation of this training scheme, the hypertrophic response was stimulated in the zebrafish heart, suggesting that the training scheme in this study may be the training intensity that can induce a physiological hypertrophic response in the zebrafish heart.

The increase in the capillary network in proportion to cardiomyocyte growth is another anatomical feature of physiologically hypertrophic heart, which is adopted to provide the myocardium with sufficient nutrients and oxygen [31, 32]. VEGF and hypoxia-inducible factors have been reported as crucial angiogenic molecules for regulating angiogenesis [33, 34]. Exercise could induce VEGF expression, activate VEGF angiogenesis signal, improve cardiac angiogenesis in elderly mice, and promote physiological heart growth and regeneration [35–37]. Furthermore, the upregulation of vegf and hif1a by swimming train was suggested to be associated with zebrafish skeletal muscle angiogenesis [31]. Compared with the control, the expression of *vegf* and *hif1a* mRNA, and the immunohistochemical expression of vegf protein were significantly upregulated in the hearts of exercise zebrafish, implying that our aerobic training program induced the angiogenesis in the heart of adult zebrafish to protect the cardiomyocyte from nutrient starvation and anoxia.

Cardiac hypertrophy increases contractility to improve the efficiency of oxygen transport and energy metabolism [38–40]. As the energy factories of the cell, the shape and location of mitochondria reflect the energy needs of cells. The mitochondria of human muscle cells enlarged and the density of internal cristae increased after long-term exercise training [41, 42]. Indeed, we observed not only increased numbers of mitochondria and cristae but also enlarged mitochondria in the heart of the training zebrafish group. Moreover, the mRNA expression of mitochondrial biogenesis factors such as *prkaa1*, *ppargc1a*, *nrf1*, and *tfeb* increased significantly, indicating that exercise training stimulated the mitochondrial synthesis in zebrafish cardiomyocytes.

In addition to mitochondrial regeneration, exercise has also been found to contribute to the elimination of damage and dysfunction of the mitochondria and maintenance of mitochondrial homeostasis in cardiomyocytes [43] via activating mitochondrial selective autophagy, which is governed by mitochondrial fusion and fission. To complete these challenges such as ROS scavenging and reversal of damage to mitochondrial DNA (mtDNA) and proteins, Drp1 dependent mitochondrial fission through the mediation of Fis1 and Mffa could to a large extent remove the damaged mitochondrial content by fission-mediated mitophagy [44]. Then, following mitochondrial fusion event regulated by mfn2 [45, 46], mfn1 and opa1 act as a compensatory process for the recycling of proteins, lipids, and mtDNAs. Mitochondrial fusion–fission dynamics play a crucial role for mitochondrial bioenergetics and quality control to maintain energy supply in response to stress [47]. Interestingly, we found that the mitochondrial dynamics factors such as drp1, fis1, mffa, mfn2 were upregulated and the mitophagy factor such as ulk1, prkaa1, foxo3a, and fundc1, were also upregulated, suggesting the mitochondrial fusion and fission cycle in exercise-trained zebrafish cardiomyocytes are more frequent to support stronger myocardial contractility.

The activation of AMP-activated protein kinase (AMPK) and antioxidation may be another adaptive physiological change to enhances mitochondrial biogenesis and energy metabolism in the present study [48]. Under low cellular ATP levels, AMPK actively stimulates various pathways, including increased glucose utilization, mobilization of lipid stores [49, 50], and physiological level of autophagy, and activates PGC1α to increase mitochondrial biogenesis [51]. Consistently, we showed the AMPK and PGC1α were upregulated in the heart of the swimming zebrafish group. Moreover, after aerobic exercise, the mRNA expression of *acsl1b*, *acsl2*, and *acads*, which are rate-limiting enzymes of fatty acids β-oxidation in the mitochondria, also increased. However, the accumulation of ROS caused by β-oxidation is not conducive to cell homeostasis. The upregulated expression of antioxidant factors such as sesn1, nrf2, hmox1a, and SOD in the myocardium may prevent this adverse effect [52, 53]. In the future, to design precise personnel exercise prescription it would be interesting to investigate the specific dose–response relationship between exercise and cardioprotective factors.

## Conclusions

Here, we successfully generated a “sports-type” myocardial hypertrophy zebrafish model, and the relevant cardioprotective responses have been explored (Fig. 6). We demonstrate that 4 weeks of aerobic exercise can induce physiological cardiac hypertrophy in zebrafish. In addition, the underlying cardiac protective mechanism is unveiled from the perspective of energy supply balance. Our experimental results provide molecular detection indicators for the sports training monitoring process of human beings, especially for the intensive sports training athletes, and provide a basis for the formulation of scientific and reasonable sports training programs.

### Supplementary Information

**Additional file 1: Table S1.** List of primers used for RT-qPCR. **Figure S1**. Effects of 80%, 100%, and 120% *U*_opt_ exercise protocols on the heart of zebrafish. **Figure S2.** Zebrafish exercise training device. **Figure S3.** Changes in body length and weight of zebrafish before and after exercise. **Figure S4.** Original western blot images of Fig. 2. **Figure S5.** Original western blot images of Fig. 4. **Figure S6.** Original western blot images of Fig. 5.

## Data Availability

All data generated or analyzed during this study are included in this published article and its Additional file.

## Abbreviations

AMR: Active metabolic rate
BL: Body length
COT: Cost of transport
CT: Calculate the threshold
H&E: Hematoxylin & eosin
ROS: Reactive oxygen species
