# Supplementary material for: Cardioprotective responses to aerobic exercise-induced physiological hypertrophy in zebrafish heart
Source: J Physiol Sci. 2021 Nov 8;71:33. doi: 10.1186/s12576-021-00818-w (PMC10717721; doi:10.1186/s12576-021-00818-w)
Supplement: Supplementary file 1 — Additional file 1: Table S1. List of primers used for RT-qPCR. Figure S1. Effects of 80%, 100%, and 120% Uopt exercise protocols on the heart of zebrafish. Figure S2. Zebrafish exercise training device. Figure S3. Changes in body length and weight of zebrafish before and after exercise. Figure S4. Original western blot images of Fig. 2. Figure S5. Original western blot images of Fig. 4. Figure S6. Original western blot images of Fig. 5. [file 12576_2021_818_MOESM1_ESM.docx]

**Additional file 1**

**Cardioprotective responses to aerobic exercise-induced physiological hypertrophy in zebrafish heart**

**Table S1**. List of primers used for RT-qPCR

| Gene | Accession number | Forward primer (5′–3′) | Reverse primer (5′–3′) |
| --- | --- | --- | --- |
| *igf1* | NM_131825.2 | ACTGGTGCTGTGCGTCCTC | GGTCCATATCCTGTCGGTTT |
| *igf1rb* | NM_152969.1 | TGCCAGACTACGACCGATTC | ACATGCTGACAGACACACCAT |
| *akt1* | NM_001281801.1 | TCGGCAGGTGTCTTCTCAAT | ACCCATTGCCATACCACGAG |
| *pik3ca* | XM_009306176.3 | CGCAATGAGAGGATGAGCGA | ACGCTGTCACGATGGAACAA |
| *mtor* | NM_001077211.2 | GCCATTCAGATCATCAACCGAGT | GCGATGCCGATTCCCACTCT |
| *nppa* | NM_198800.3 | TTTGGCAGCAGACGGATGTA | TCTGATGCCTCTTCTGTTGCC |
| *myh7* | NM_001112733.1 | TGGTGAGGGAGGAAAGAGCAT | CGCAGAATCTTACCCTCCTCG |
| *cebpb* | NM_131884.2 | ATGACATGCGCTCGTACCTG | TGTACTCGTCGCTGTCCTTG |
| *rps6kb1b* | NM_213076.1 | TGTTAACCGGAGCACCTCCT | GTCCCTGGCTTCTTGTGTGA |
| *eif4ba* | NM_001099237.2 | AGAGCGAGATCTCAGACCAG | CCATCAGCAAGGCTGCGTAT |
| *srfa* | NM_001110526.1 | ATCCTCAGTGGCCATGCAC | AAGAGAAGCGCCTGGCAT |
| *mapk1* | NM_182888.2 | CAGACCCTAAAGCGCTGGAC | GTCGAACTTGAACGGAGCCT |
| *mapk3* | NM_201507.1 | ATGCTTAACTCCAAGGGCTACAC | GCTGGTCCAGATAGTGCTTCC |
| *hif1ab* | NM_001310042.1 | GCTCGTCCACAGAACAGGAT | AGCGCAGTGAAGAACCTTCC |
| *vegfc* | NM_205734.1 | AAGCAGATGCCATGCAGGAG | CATCCACACTACCCGCTGAA |
| *nrf1* | NM_001328540.1 | GCACTGAAGTTTGGTGAAGCA | CCGGAACTCCAGTTAACGCT |
| *tfam* | NM_001077389.1 | AGTGCATCTGTTGTGAGGTGT | TCCACTGCTGGGCAATCTTT |
| *mffa* | NM_200536.1 | AGAAACGACTCTGCGTTTGC | TGGCTCGCTCTTTGTTCTCC |
| *fis1* | NM_001310843.2 | GGACCCGTTTCCAGAGACAC | TGCTTGTGTGAACCAGTTCTTC |
| *cited4a* | NM_001044982.2 | CACCCACTTGGCCCAATGAA | TGAACTCGATCCAGCCCAAG |
| *sirt1* | XM_005173034.3 | CCTCAGCCACAGATTGGGTC | TCTGGAGGAAGCACCGTTTC |
| *mfn1b* | NM_200647.1 | GGAACTGATGTGACCACCGA | TGGAAGAAATGCTTCTCCGTGT |
| *mfn2* | NM_001128254.2 | CATCCTTCACCAGTCGTGCT | GGCTTCAGACCGTCGATCAT |
| *fundc1* | NM_001002711.2 | AGGCATCAATGGTGGAGTCG | AGATATCCAGCACACCATCCG |
| *foxo3a* | NM_001009988.1 | GAGCAGTGATGAACAGGAA | TTGATGAGGTTGACGATGG |
| *ulk1a* | NM_001130631.1 | GCCTCAGTCCCAGTATTCCAC | CACCGGCACAGGAGATGATT |
| *acads* | NM_001003743.1 | GCAGCAGGAGGGAAACGAA | GAGGCATCGGCACTAGGAA |
| *acsl1b* | NM_001003569.2 | AAGAAAGTGCAGGCCAGTGT | TGCTGTCCAGTCTCCTGGTA |
| *acsl2* | XM_002663239.6 | AGAGAGGACTGCGGATAGCA | TGCAATGACCCACTCAGGTC |
| *mt-nd1* | NP_059331.1 | AGCCATCTCAAGCCTAGCAG | ATTGTTTGCGCTACAGCTCG |
| *mt-nd2* | NP_059332.1 | GACCTACCAGCCACAGCTAC | TTGGGTCGTTTGTACCCGTC |
| *mt-nd3* | NP_059338.1 | ACCACTCCCATGAGGAGATCA | CTTGGGCTCATTCGTAGGCT |
| *sod1* | NM_131294.1 | GCCAGTGAAGGTGACTGGTG | GTCTCCGACGTGTCTCACA |
| *sod2* | NM_199976.1 | GATATGTTCGGAGGTGCGCT | ATGTTGCATGGTGCTTGCTG |
| *sesn1* | NM_001002660.1 | GCGTTCAATCTGACATACAAC | CCATAATCATAATCGTCGTACC |
| *nfe2l2a* | NM_182889.1 | CAGAGTTGCAGCAGTGCCT | TGGTGCTTCTGTGGAAGGTT |
| *hmox1a* | NM_001127516.1 | TGGACAGAAACGCAGACCAC | GACTGCTCTTGCCAATCTCTCT |
| *tfeb* | NM_001257192.1 | ACTCCTGCCCTGCGAATCTA | CAGCGTACATCCAGGTCGTT |
| *bnip3* | NM_001012245.1 | GCCTACCATGTGACAGTCCT | TCGACCAGTCCCAGATCCAA |
| *aldh4a1* | NM_201158.1 | GAGCCCACAATAATCGAGAC | CAGATTTGTCTTGGGGGAAG |
| *prkab1a* | NM_001002632.1 | TGCGAAGGAAGAACCCAGTC | AGACGCTTCGCCTTTTGCT |
| *ppargc1a* | XM_017357139.2 | GCGAGGGAACGAGTGGATTT | CTCTCCACACCGAATCCTGA |
| *gapdh* | NM_001115114.1 | ATCATCTCTGCCCCAAGTGC | ACGGTCTTCTGTGTTGCTGT |
| *cox4i1* | NM_214701.1 | GGCAACTACGGCATTTCGTC | CGACCTTCGCAACTCCATGT |
| *dnm1l* | NM_200922.1 | AAACAGCTGAACTGTGTGGTG | GTAAGTCCGCCAAGAGGATCG |

**Abbreviations***: igf1, insulin-like growth factor 1; igf1rb, insulin-like growth factor 1b receptor; akt1, v-akt murine thymoma viral oncogene homolog 1; pik3ca, phosphatidylinositol-4 5-bisphosphate 3-kinase catalytic subunit alpha; mtor, mechanistic target of rapamycin kinase; nppa, natriuretic peptide A; myh7, myosin heavy chain 7; cebpb, CCAAT enhancer-binding protein beta; rps6kb1b, ribosomal protein S6 kinase b polypeptide 1b; eif4ba, eukaryotic translation initiation factor 4Ba; ppargc1a, peroxisome proliferator-activated receptor-gamma coactivator 1 alpha; cited4a, Cbp/p300-interacting transactivator with Glu/Asp-rich carboxy-terminal domain 4a; srfa, serum response factor a; mapk1, mitogen-activated protein kinase 1; mapk3, mitogen-activated protein kinase 3; hif1ab, hypoxia-inducible factor 1 subunit alpha b; vegfc, vascular endothelial growth factor c; nrf1, nuclear respiratory factor 1; tfam, transcription factor A mitochondrial; mffa, mitochondrial fission factor a; fis1, fission mitochondrial 1; sirt1, sirtuin 1; mfn1b, mitofusin 1b; mfn2, mitofusin 2; fundc1, FUN14 domain containing 1; tfeb, transcription factor EB;* *bnip3, BCL2 interacting protein 3; dnm1l, dynamin 1-like; foxo3a, forkhead box O3A; ulk1a, unc-51 like autophagy activating kinase 1a; acads, acyl-CoA dehydrogenase short-chain; acsl1b, acyl-CoA synthetase long chain family member 1b; acsl2, acyl-CoA synthetase long-chain family member 2; mt-nd1, NADH dehydrogenase 1; mt-nd2, NADH dehydrogenase 2; mt-nd3, NADH dehydrogenase 3; sod1, superoxide dismutase 1 (soluble); sod2, superoxide dismutase 2 (mitochondrial); sod1, superoxide dismutase 1 (soluble); sod2, superoxide dismutase 2 (mitochondrial); sesn1, sestrin 1; nfe2l2a, nuclear factor erythroid 2-like 2a; hmox1a, heme oxygenase 1a; aldh4a1, aldehyde dehydrogenase 4 family member A1; prkaa1, protein kinase AMP-activated alpha 1 catalytic subunit; gapdh, glyceraldehyde-3-phosphate dehydrogenase; cox4i1, cytochrome c oxidase subunit 4I1*


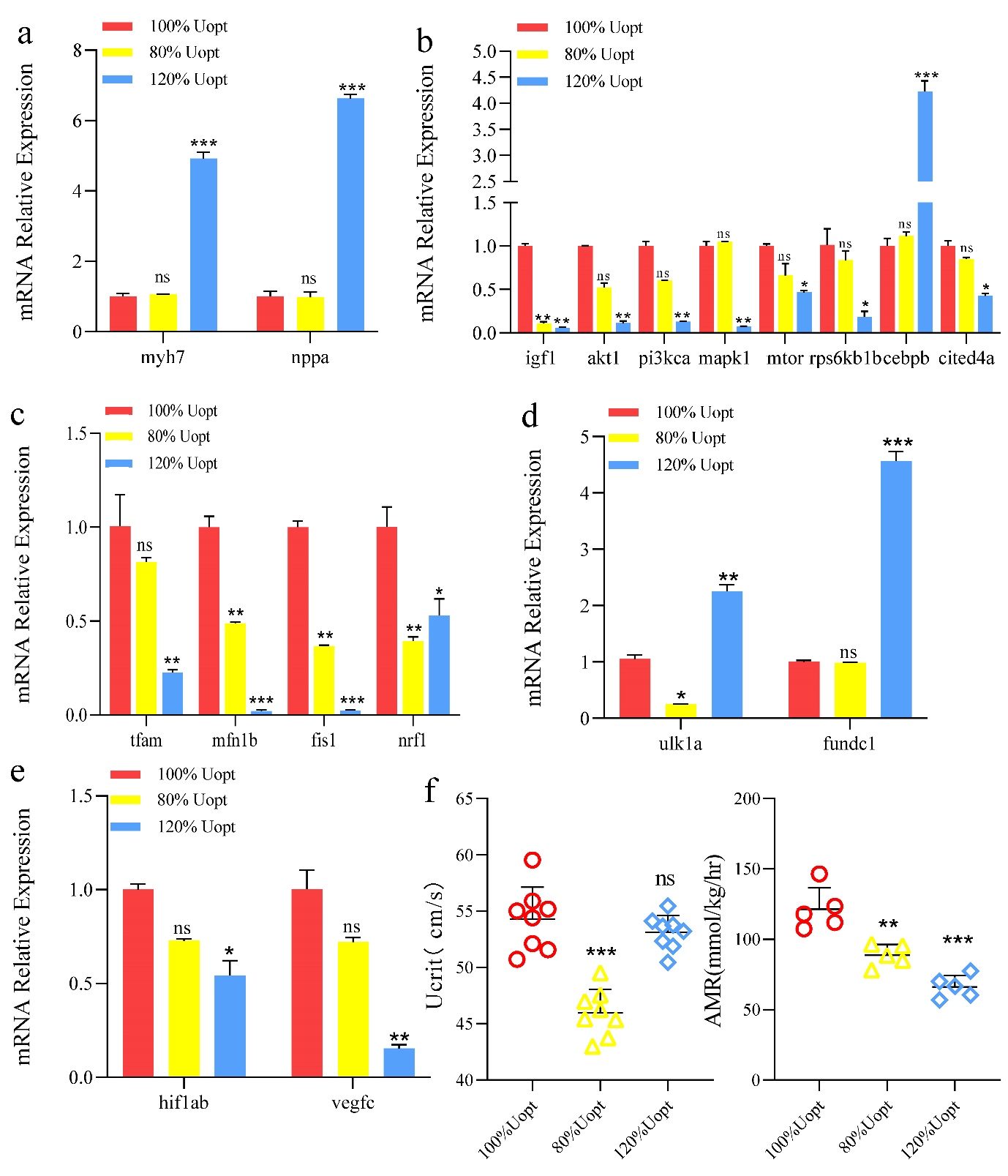


**Figure S1**. Effects of 80%, 100%, and 120% U_opt_ exercise protocols on the heart of zebrafish. (a) Expression of mRNA for the cardiac pathological markers myh7 and nppa in zebrafish under three different intensity exercise regimens. (b) mRNA expression of genes related to cardiac protein synthesis and proliferation in zebrafish under three different intensity exercise regimens. (c) mRNA expression of genes related to mitochondrial fission and fusion in zebrafish heart under three different intensity exercise regimens. (d) mRNA expression of autophagy-related genes in zebrafish heart mitochondria under three different intensity exercise regimens. (e) mRNA expression of cardiac angiogenesis-related genes in zebrafish under three different intensity exercise regimens. (f) Effects of U_crit_ and AMR in zebrafish under three different intensity exercise regimens. Statistical significance was determined using the Student’s t-test: **p* < 0.05; ***p* < 0.01; ****p* < 0.001. ns, not significant.


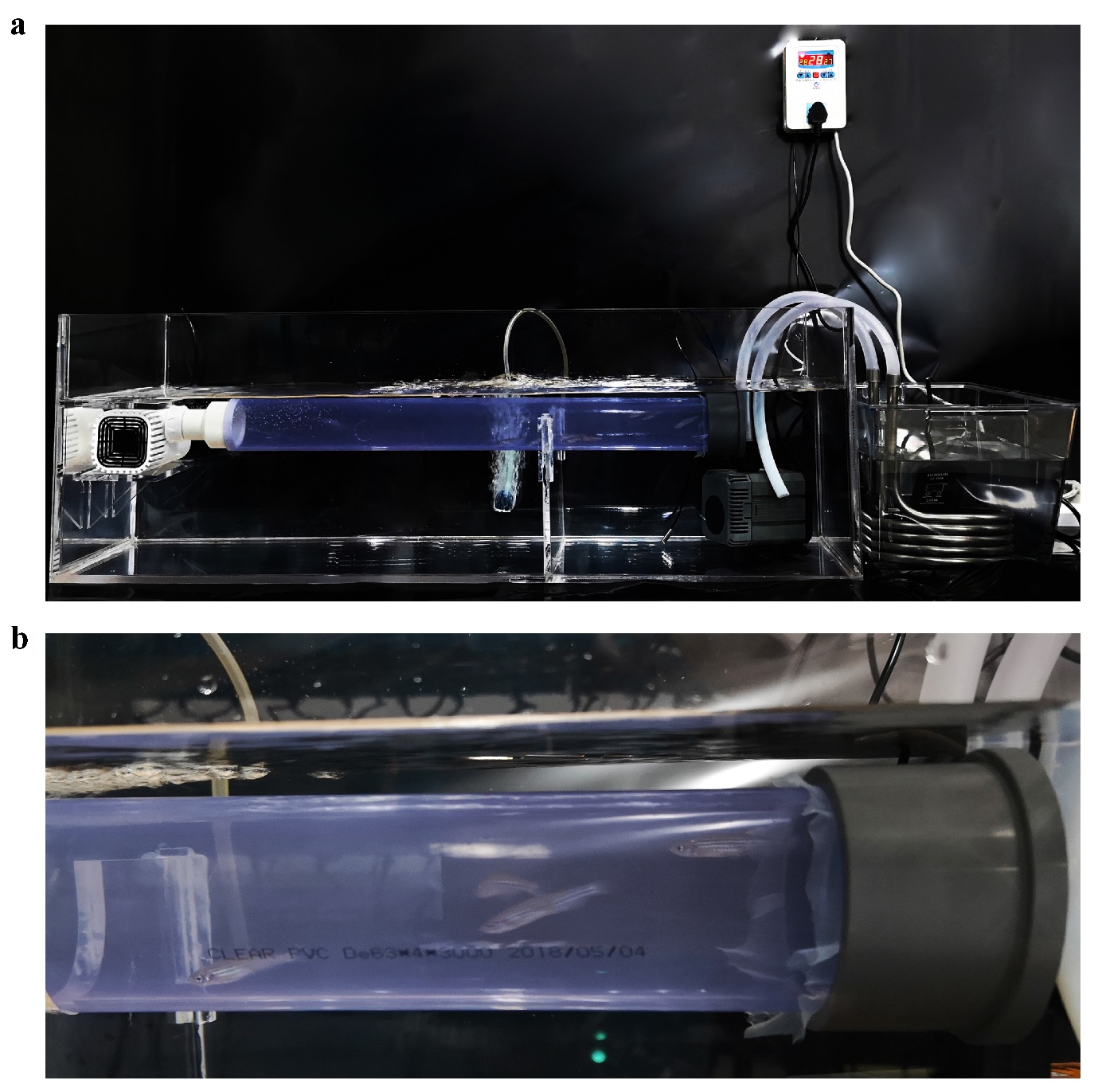


**Figure S2.** Zebrafish exercise training device (Patent NO. ZL 2019216725786). (a) Overall view of the zebrafish exercise unit, including the water tank, inverter pump, swim lane, heating system, and oxygenation pump. (b) Zebrafish swimming in the training device.


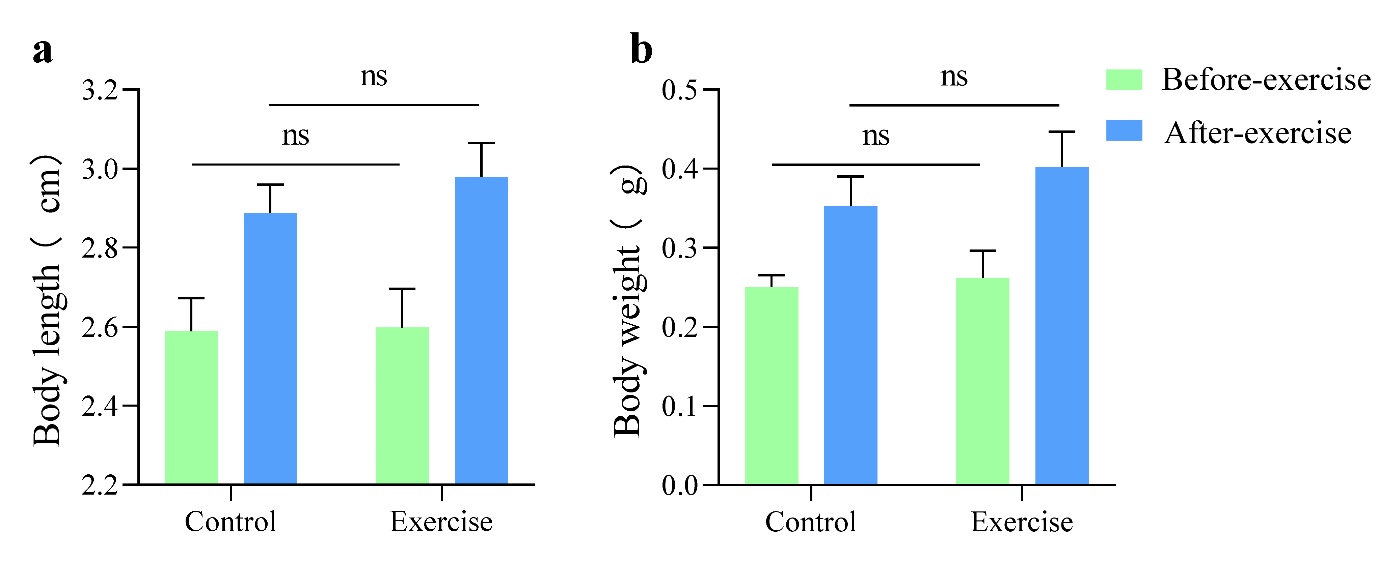


**Figure S3.** Changes in body length and weight of zebrafish before and after exercise. (a) The body length of zebrafish before and after exercise. (b) The bodyweight of zebrafish before and after exercise. Statistical significance was determined using the Student’s t-test: ns, not significant.


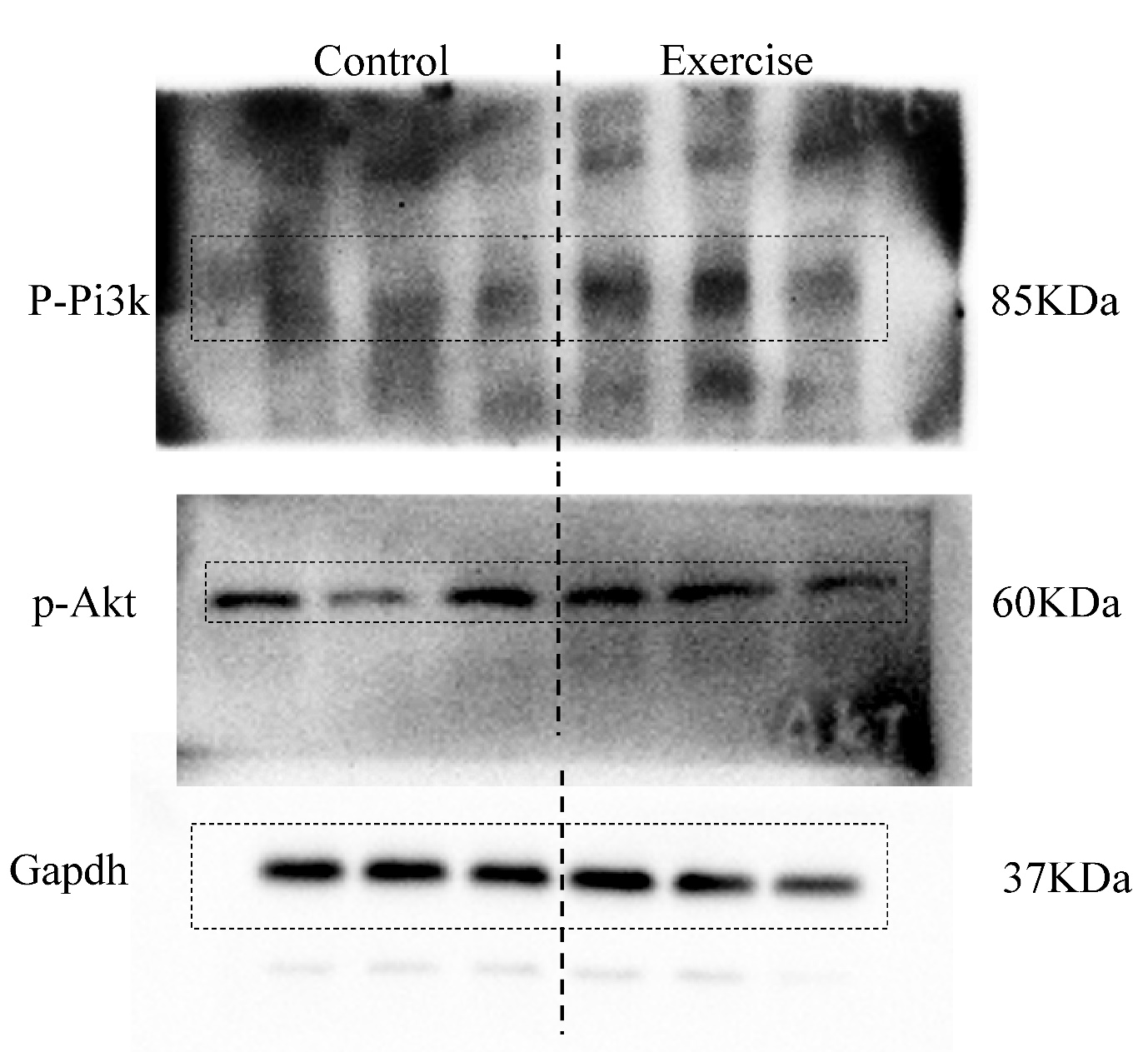


**Figure S4.** Original western blot images of Figure 2.


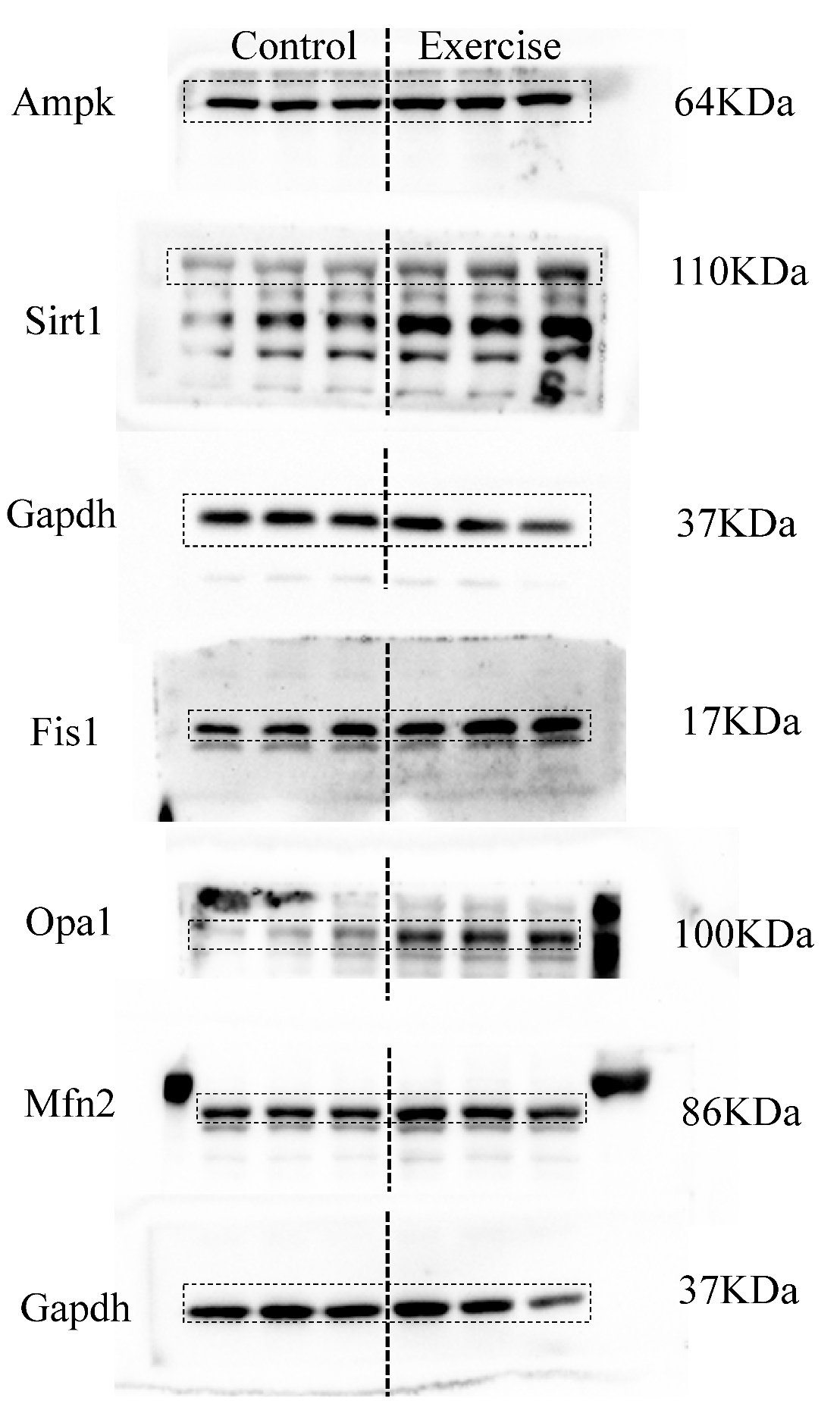


**Figure S5.** Original western blot images of Figure 4.


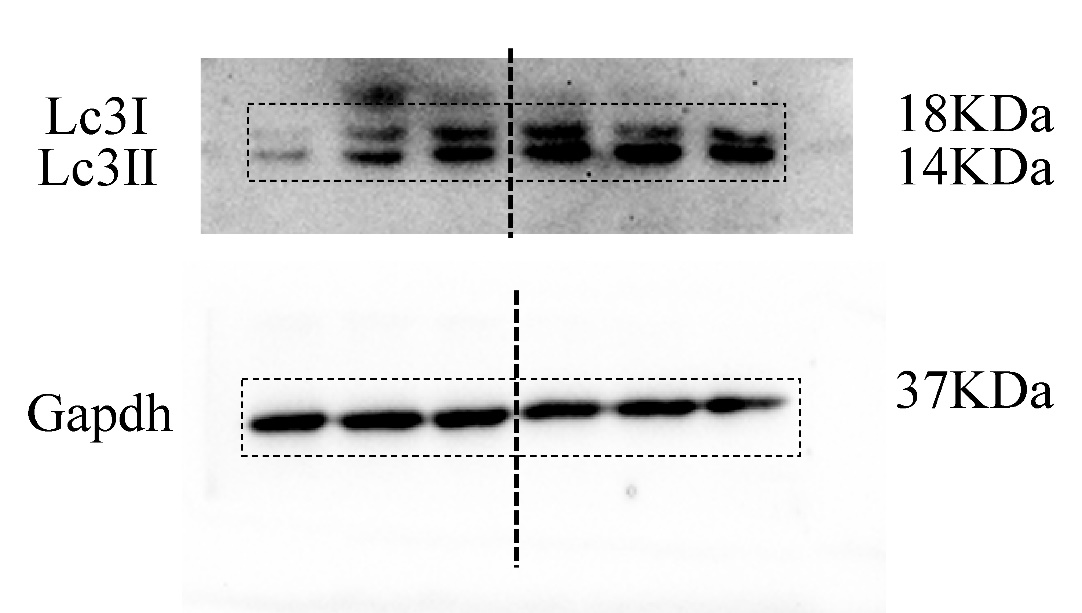


**Figure S6.** Original western blot images of Figure 5.
